# FoxO3a Drives the Metabolic Reprogramming in Tamoxifen-Resistant Breast Cancer Cells Restoring Tamoxifen Sensitivity

**DOI:** 10.3390/cells12242777

**Published:** 2023-12-06

**Authors:** Marco Fiorillo, Elena Ricci, Mariarosa Fava, Camilla Longobucco, Federica Sotgia, Pietro Rizza, Marilena Lanzino, Daniela Bonofiglio, Francesca Luisa Conforti, Stefania Catalano, Ines Barone, Catia Morelli, Saveria Aquila, Michael P. Lisanti, Diego Sisci

**Affiliations:** 1 Department of Pharmacy, Health and Nutritional Sciences, University of Calabria, 87036 Rende, Italy; marco.fiorillo@unical.it (M.F.); riccielena91@gmail.com (E.R.); mariarosa.fava@unical.it (M.F.); camilla.longobucco@unical.it (C.L.); pietro.rizza@unical.it (P.R.); marilena.lanzino@unical.it (M.L.); daniela.bonofiglio@unical.it (D.B.); francescaluisa.conforti@unical.it (F.L.C.); stefania.catalano@unical.it (S.C.); ines.barone@unical.it (I.B.); diego.sisci@unical.it (D.S.); 2 Translational Medicine, School of Environment and Life Sciences, Biomedical Research Centre (BRC), University of Salford, Greater Manchester M5 4WT, UK; fsotgia@gmail.com (F.S.); michaelp.lisanti@gmail.com (M.P.L.)

**Keywords:** FoxO3a, tamoxifen resistance, breast cancer, glycolysis, cancer metabolism

## Abstract

Tamoxifen-resistant breast cancer cells (TamR-BCCs) are characterized by an enhanced metabolic phenotype compared to tamoxifen-sensitive cells. FoxO3a is an important modulator of cell metabolism, and its deregulation has been involved in the acquisition of tamoxifen resistance. Therefore, tetracycline-inducible FoxO3a was overexpressed in TamR-BCCs (TamR/TetOn-AAA), which, together with their control cell line (TamR/TetOn-V), were subjected to seahorse metabolic assays and proteomic analysis. FoxO3a was able to counteract the increased oxygen consumption rate (OCR) and extracellular acidification rate (ECAR) observed in TamR by reducing their energetic activity and glycolytic rate. FoxO3a caused glucose accumulation, very likely by reducing LDH activity and mitigated TamR biosynthetic needs by reducing G6PDH activity and hindering NADPH production via the pentose phosphate pathway (PPP). Proteomic analysis revealed a FoxO3a-dependent marked decrease in the expression of LDH as well as of several enzymes involved in carbohydrate metabolism (e.g., Aldolase A, LDHA and phosphofructokinase) and the analysis of cBioPortal datasets of BC patients evidenced a significant inverse correlation of these proteins and FoxO3a. Interestingly, FoxO3a also increased mitochondrial biogenesis despite reducing mitochondrial functionality by triggering ROS production. Based on these findings, FoxO3a inducing/activating drugs could represent promising tools to be exploited in the management of patients who are refractory to antiestrogen therapy.

## 1. Introduction

The involvement of the estrogen receptor α (ER) in the development and progression of breast cancer (BC) has been confirmed by numerous ‘in vivo’ and ‘in vitro’ studies that have elucidated many aspects of ER regulation and functions, paving the way to the wide range of endocrine therapies commonly used in clinics for the treatment of ER-positive (ER+) BC patients [1,2,3]. The use of ‘selective ER modulators’ (SERMs) or ’degraders’ (SERDs), such as tamoxifen (Tam) and fulvestrant, respectively, as well as of aromatase inhibitors (AIs), e.g., anastrozole, significantly improved both the relapse-free survival (RFS) and the overall survival (OS) of ER + BC patients. However, many patients acquire resistance to these endocrine therapies, making the treatment ineffective [4]. The potential mechanisms underlying the evolution toward anti-estrogen-resistant phenotypes have been attributed to various causes, including the ligand-independent activation of receptors [5], the induction of growth-activating pathways [6,7], and the perturbation of normal metabolism [8]. Significant alterations in intermediary metabolism have been found in most tumors, including BC. Deep alterations in glycolysis, mitochondrial oxidative phosphorylation, and lipid and amino acid metabolism have been described in BC cells (BCCs) [9]. Moreover, the metabolic phenotype of BCCs changes as they progress from non-metastatic to metastatic [10]. We have previously defined the basal metabolic profile and the metabolic alterations induced by the acquired resistance to Tam treatment of MCF-7 BCCs [8]. The metabolic phenotype of Tam-resistant MCF-7 cells (TamR) is characterized by increased mitochondrial biogenesis, increased ATP production and a reduction in reduced glutathione levels, indicating the adaptation of cell metabolism to the acquired cell functions. The growth and viability of malignant cells are strongly dependent on their ability to adopt an altered metabolic profile that fulfills their bioenergetic requirements, a condition frequently stemming from the promotion of glucose-dependent survival through the Akt signal transduction pathway [11]. ATP generation through glycolysis instead of oxidative phosphorylation, even under normal oxygen concentrations, was the earliest described metabolic phenotype observed in tumor cells (the Warburg effect) [12]. In this regard, glycolysis provides cancer cells not only with energy but also with carbon sources that provide anabolic precursors for biosynthesis [13]. However, complex interplay between glucose metabolism and mitochondrial activity exists and it depends on several factors, such as the region of the tumor (whether hypoxic or non-hypoxic) and the tumor microenvironment. Depending on the combinations of these factors and a given cellular context, cancer cells can manifest an array of metabolic phenotypes, ranging from the predominance of the glycolytic phenotype to a glycolytic partial phosphorylative and/or phosphorylative phenotype. In this context, the Forkhead box class O (FoxO)3a, an Akt downstream target, has been described as an important modulator of cell metabolism. Indeed, FoxO3a has been shown to reduce glycolysis through the transcriptional induction of tuberous sclerosis complex 1 (TSC1) tumor suppressor that opposes mTOR Complex 1 (mTORC1) activation [14]. In addition, in a low-glucose regimen, FoxO3a has been found to bind to mitochondrial DNA regulatory regions by forming a complex containing the NAD-dependent mitochondrial deacetylase sirtuin-3 and the mitochondrial RNA polymerase, causing an increase in mitochondrial respiration [15]. Moreover, FoxO3a inactivation has been reported to lead to intracellular ROS accumulation [16], with consequent oxidative stress due to mitochondrial dysfunction. On the other hand, FoxO3a activation has been reported to induce autophagy, initially as a protective attempt to save energy to survive, while leading to cell death under persistent stress conditions [17]. Therefore, being involved in the regulation of a wide spectrum of cellular processes, including metabolism, autophagy and response to stress stimuli [18], the pharmacological modulation of FoxO3a seems to hold a great therapeutic potential. In this context, we recently reported how FoxO3a results downregulated in TamR cells and how its re-expression is able to restore the apoptotic response to Tam treatment [19].

Here, we add a new dowel to our understanding of the role of FoxO3a in reverting the resistance to Tam in BCCs, demonstrating how active FoxO3a affects cell metabolism by decreasing their glycolytic rate and mitochondrial oxygen consumption in TamR cells. 

## 2. Materials and Methods

### 2.1. Generation of Tam Resistant Cell Lines

ERα+ human breast cancer epithelial cell lines MCF-7, T-47D and ZR-75-1 (ZR-75) were purchased from ATCC (LGC Standards S.r.l., Milan, Italy), and authenticated and cultured as previously described [20]. 

Tam-resistant MCF-7 (MCF-7/TR), T-47D (T-47D/TR) and ZR-75 (ZR-75/TR) cell lines were obtained after long-term cultivation of parental cells in their own growing medium, with increasing concentrations of Tam (Merck, Milan, Italy), followed by chronic exposure to Tam 1 μM, as previously reported [19]. 

### 2.2. FoxO3a Inducible Stable Cell Lines

TamR/TetOn-AAA cell line and the relative control TamR/TetOn-V were developed as previously described [19]. Briefly, TamR cells were subjected to a first transfection with the pTet-On regulator plasmid, carrying the G418 resistance. Antibiotic-resistant TamR/TetOn cells were further transfected with the pTRE-F3aAAA plasmid, bearing a cDNA encoding a constitutively active form of the human FoxO3a gene, a GFP-encoding cassette and the Zeocin resistance gene. Control cell lines (TamR/TetOn-V) were established using the same protocol, but the pTRE backbone (vector only) was used in place of the F3aAAA cDNA insert. Pools of TamR/TetOn-V and TamR/TetOn-AAA cells were maintained in DMEM/F12 containing G418 and Zeocin selection antibiotics, plus Tam 1 μM. The tetracycline derivative Doxycycline (Dox) (Sigma-Aldrich, Merck, Milan, Italy) was employed at a concentration of 1 μg/mL to induce F3aAAA expression.

### 2.3. Plasmids and Transient Transfections 

To transiently over-express FoxO3a, the 1319 pcDNA3 flag FKHRL1AAA (F3aAAA), encoding a constitutively active triple mutant of FoxO3a (Addgene, plasmid 10709), was transfected with FuGENE^®^ HD (Promega Italia S.r.l, Milan, Italy). pcDNA3.1 vector (Invitrogen) was used as control (pcDNA3). 

### 2.4. RNA Extraction, Reverse Transcription, and Real-Time (RT)-PCR

Total RNA was extracted with TRIzol reagent (Thermo Fisher Scientific Inc., Waltham, MA, USA). Two µg was used for reverse transcription using the High-Capacity cDNA Reverse Transcription Kit (Thermo Fisher Scientific Inc.) according to the manufacturer’s instructions. cDNA was mixed to SYBR green Universal PCR Master Mix (Bio-Rad, Milan, Italy) and subjected to RT-PCR in an iCycler iQ (Bio-Rad, Milan, Italy). The primer sequences used in this study are listed in Table S1. The samples were normalized on the relative 18S rRNA content. The results were reported as n-fold differences in gene expression vs. controls.

### 2.5. Western Blotting (WB)

Cytosolic proteins were obtained using a lysis buffer containing 50 mM HEPES (pH 7.5), 150 mM NaCl, 1% Triton X-100, 1.5 mM MgCl_2_, 10 mM EGTA (pH 7.5), 10% glycerol, and inhibitors (0.1 mM Na_3_VO_4_, 1% PMSF and 20 mg/mL aprotinin). All reagents were from Merck, Milan, Italy. After separation on SDS-PAGE gel and transfer to nitrocellulose membranes, proteins were detected with specific antibodies: FoxO3a (75D8, #2497) (from Cell Signaling Technology, Danvers, MA, USA), PFKM (MAB7687; Clone 842736) and, LDHA (MAB9158; Clone 2066C) (from R&D Systems, Minneapolis, MN, USA), Aldolase A (1C5B2) (from Novus Biologicals, Centennial, CO, USA), β-Actin (AC-15) (Sigma-Aldrich, Merck, Milan, Italy). IRDye secondary Abs, the Odyssey FC Imaging System imager and the Image Studio™ Lite v5.2 software were all from LI-COR Biosciences GmbH, Bad Homburg, Germany.

### 2.6. Glucose Assay

Glucose content was determined by using an Inter-Medical kit (Biogemina Italia Srl, Catania, Italy). Glucose oxidase catalyzes the oxidation of glucose to gluconic acid, generating H_2_O_2_ which is detected by using a chromogenic oxygen acceptor, phenol, 4-amino-phenazone in the presence of peroxidase. The color intensity observed is proportional to the glucose concentration in the sample. Data are presented as nmol/mg protein.

### 2.7. Lactate Dehydrogenase (LDH) Activity Assay 

Cell proteins were extracted and counted as previously described [21]. Lysates were used to evaluate LDH activity according to the Inter-Medical kit protocol (Biogemina Italia Srl, Catania, Italy) [22]. Absorbance measures at 490 nm were normalized on protein content and reported on a graph.

### 2.8. Glucose-6-Phosphate Dehydrogenase (G6PDH) Activity Assay

The conversion of NADP+ to NADPH, catalyzed by G6PDH, was measured by examining the increase in absorbance at 340 nm. Briefly, after treatment, 50 µg of proteins was diluted in 1 mL of buffer containing 100 mM MgCl_2_, 100 mM triethanolamine, 10 mg/mL NADP+, pH 7.6 (Merck, Milan, Italy) and 10 mg/mL glucose-6-phosphate) for spectrophotometric determination. The absorbance of samples was read at 340 nm every 20 s for 1.5 min. Data are presented as nM/min/mg protein.

### 2.9. Seahorse XFe96 Metabolic Flux Analysis

Real-time oxygen consumption rates (OCRs) and extracellular acidification rates (ECARs) in all transfected cells treated with Dox 1 µg/mL for 48h and 72h were determined using the Seahorse Extracellular Flux (XFe96) analyzer (Agilent, Santa Clara, CA, USA). Briefly, 1 × 10^4^ cells per well were seeded into XFe96 well cell culture plates and incubated overnight to allow cell attachment. Then, cells were treated with Dox 1 µg/mL for 48 h and 72 h. Empty vector (Vector) control cells were processed in parallel. After 48 or 72 h hours of incubation, cells were analyzed following the manufacturer’s protocol. Measurements were normalized by examining the protein content (Bradford assay). Data sets were analyzed using XFe96 software and GraphPad Prism software using one-way ANOVA and Student’s t-test calculations. All experiments were performed in quintuplicate, three times independently.

### 2.10. Sulfo-Rhodamine B (SRB) Assay

Cells were fixed in 10% trichloroacetic acid for 1 h at 4 °C, stained with SRB for 15 min, and washed three times with 1% acetic acid. The incorporated dye was solubilized with 10 mM Tris-HCl, pH 8.8 (all reagents were from Merck, Milan, Italy). Absorbance was spectrophotometrically measured at 540 nm in a FluoStar Omega plate reader (BMG Labtech, Ortenberg, Germany). Background measurements were subtracted from all values.

### 2.11. Label-Free Unbiased Semi-Quantitative Proteomics Analysis

Cell protein extracts were prepared for trypsin digestion via the sequential reduction of disulphide bonds with TCEP and alkylation with MMTS. Peptides were extracted and prepared for LC-MS/MS. All LC-MS/MS analyses were performed as previously reported [23]. Five replicates were analyzed for each sample. Data were analyzed using the Mascot search.

### 2.12. Mitochondrial Staining

MitoTracker Orange and MitoTracker Deep-Red (both from Thermo Fisher Scientific Inc.) were used to assess the mitochondrial activity and the mitochondrial mass, respectively. After 72 h, cells were incubated with 10 nM MitoTracker staining solution for 30–60 min at 37 °C. Cells were then washed in PBS, harvested, resuspended in PBS and analyzed using the Attune NxT Flow Cytometer [8]. 

### 2.13. Reactive Oxygen Species (ROS) Assessment

Intracellular ROS were quantified using CellROX™ Deep Red (Thermo Fisher Scientific Inc.), as previously described [24]. Briefly, cells were treated with Dox (1 µg/mL) for 48  h and 72 h and then harvested and resuspended in 5 μm CellROX™ Deep Red fluorescent dye. Stained cells were centrifuged, resuspended in PBS and then analyzed using the Attune NxT Flow Cytometer.

### 2.14. cBioPortal Analysis

Gene co-expression profiles were extracted from “Breast Cancer (METABRIC, NATURE 2012 & NAT. COMM 2016)”, using cBioPortal (cbioportal.org Cerami et al., 2012 & Gao et al., 2013). mRNA expression profiling (RNA Seq V2 RSEM) was carried out via RNA-sequencing of tissue samples derived from n = 1329 patients with breast cancer. Searching criteria: tumor type (breast carcinoma) and ER status (positive).

### 2.15. Statistical Analysis

Data were analyzed using the Student’s *t*-test and 2-way ANOVA Šídák’s multiple comparisons test, where indicated, using the GraphPad Prism 4 software program. All values with *p* < 0.05 were considered statistically significant.

## 3. Results

### 3.1. Nuclear FoxO3a Restores the Sensitivity of TamR BCCs to Tam

**Figure 1:**
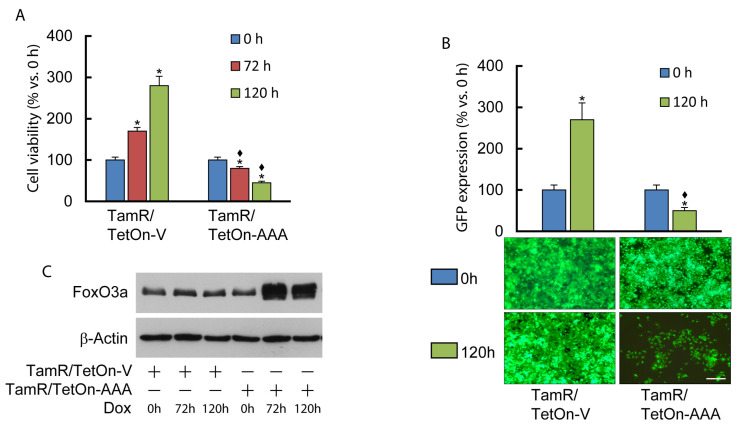
Nuclear FoxO3a restores the sensitivity of TamR breast cancer cells to Tam. TamR/TetOn-AAA and control Vector (TamR/TetOn-V) cell lines have been subjected to proliferation assay. Serum-starved cells (for 24 h) were treated with Tam 1 μM and Dox 1 µg/mL (the latter to activate Tet-induced transcription). (**A**) At the indicated time points, cells were subjected to SRB assay (see Materials and Methods). (**B**) GFP-expressing cells were counted by means of ImageJ after 0 and 120 h of Dox (1 µg/mL) treatment. Data are the mean ± SD of three independent experiments (at least in triplicate) reported as variation (%) vs. time 0 (* *p* < 0.01) or vs. the correspondent time point in TamR/TetOn cells (♦ *p* < 0.001). Images from microscope (10×) after 120 h in absence or presence of Dox 1 µg/mL. Scale bar = 10\0 μm. (**C**) A duplicate set of TamR/TetOn-V and TamR/TetOn-AAA cells were treated as in (**A**) and subjected to WB analysis to confirm Dox-dependent FoxO3a induction at 72 h and 120 h.

We previously demonstrated that FoxO3a is able to restore the sensitivity of TamR BCCs to Tam treatment [19]. Unlike the parental MCF-7 cell line, whose growth is notoriously inhibited by Tam (Ref. [19] and Figure S1A), the antiestrogen did not show any effect on the proliferation of control TamR/TetOn-AAA cells compared to the control TamR/TetOn-V cell line (Figure 1 and Figure S1B). Our previous studies also showed that the over-expression of a constitutive active form of FoxO3a (F3a-AAA) in parental MCF-7 cells as well as in TamR/TetOn-AAA cells causes a dramatic reduction in cell proliferation (Refs. [19,25] and Figure S1A,B). Notably, FoxO3a overexpression significantly improved the antiproliferative effect of Tam in parental cells and re-established the response to Tam treatment in TamR/TetOn-AAA cells (Figure 1 and Figure S1).

### 3.2. FoxO3a Counteracts the Increased Oxygen Consumption Rate and Extracellular Acidification Rate Observed in TamR BCCs

**Figure 2:**
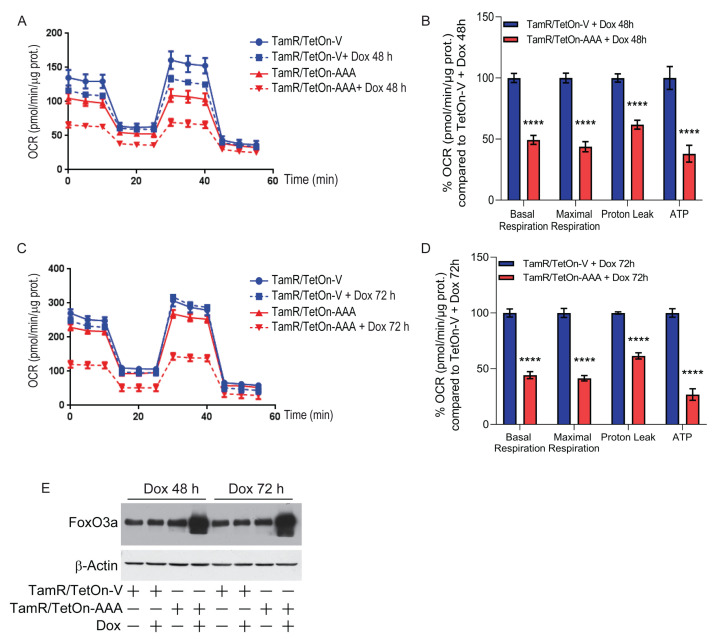
FoxO3a causes a significant decrease in mitochondrial oxygen consumption and mitochondrial ATP production in TamR BCCs. TamR/TetOn-AAA and TamR/TetOn-V cells were plated, left to attach and then treated with Dox 1 µg/mL for 48 (**A**,**B**) and 72 (**C**,**D**) hours to induce FoxO3a expression. At the end of the incubation period, the mitochondrial function was evaluated by determining the real-time oxygen consumption rates (OCR) (see Section 2). OCR was measured under basal conditions followed by the sequential addition of 10 μM Oligomycin, 9 µM FCCP, 10 µM Rotenone and 10 μM Antimycin A. Each data point represents an OCR measurement. Representative line graphs of three independent experiments, normalized vs. protein content, are shown (**A**,**C**). Histograms represent the % of basal and maximal respiration and Proton leak calculated at 48 (**B**) and 72 (**D**) hours compared to TamR/TetOn-V cells. A duplicate set of cells was plated on 60 mm dishes to assess FoxO3a overexpression by WB analysis. β-Actin was used as loading control (**E**). Data are reported as the mean ± SD of three independent experiments (at least in triplicate). Statistical significances are calculated by using 2way ANOVA Šídák’s multiple comparisons test evaluating the differences between TamR/TetOn-AAA and TamR/TetOn-V samples (**** *p* < 0.00005).

**Figure 3:**
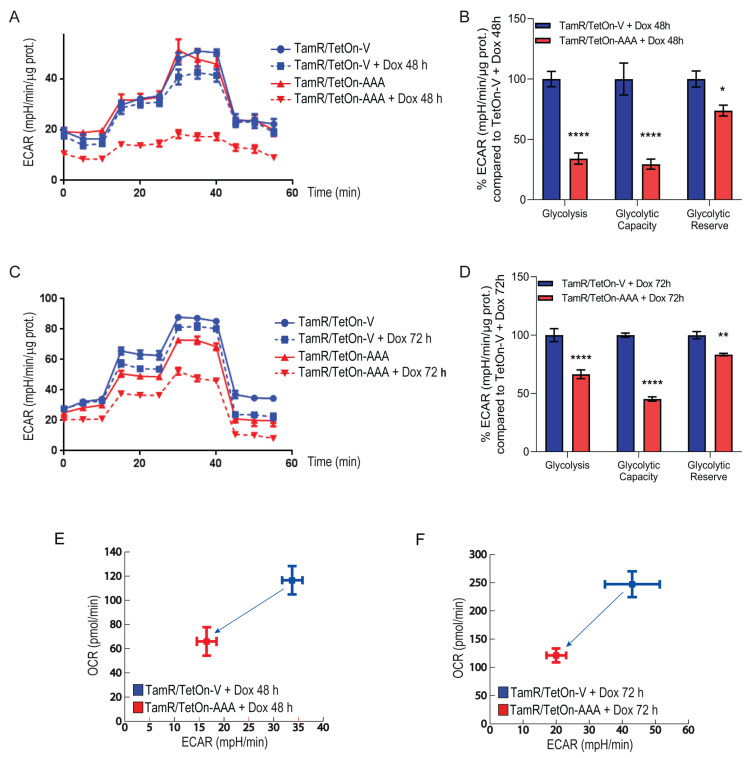
FoxO3a reduces the energetic activity of TamR BCCs, reducing their glycolytic rate. Extracellular acidification rate (ECAR) was evaluated in TamR/TetOn-AAA and TamR/TetOn-V cell clones (described in Materials and Methods). Cells were plated, left to attach and then treated with Dox 1 µg/mL for 48 (**A**,**B**) and 72 (**C**,**D**) hours to induce FoxO3a expression. Extracellular acidification rate (ECAR) was then evaluated (see Section 2). ECAR was measured under basal conditions followed by the sequential injection of 80 mM glucose, 9 µM oligomycin, and 500 mM 2-deoxyglucose. Each data point represents an ECAR measurement. Representative line graphs of three independent experiments, normalized vs. protein content, are shown (**A**,**C**). Histograms represent the % of glycolysis, glycolytic capacity, glycolytic reserve of TamR/TetOn-AAA cells calculated at 48 (**B**) and 72 (**D**) hours, compared to TamR/TetOn-V cells. OCR/ECAR ratios at 48 h and 72 h are reported in (**E**,**F**), respectively. FoxO3a and **β**-Actin expression are shown in Figure 2E. Data are reported as the mean ± SD of three independent experiments (at least in triplicate). Statistical significances are calculated by using 2way ANOVA Šídák’s multiple comparisons test evaluating the differences between TamR/TetOn-AAA and TamR/TetOn-V samples (* *p* < 0.05, ** *p* < 0.005, **** *p* < 0.00005).

TamR BCCs have been previously described to be characterized by an enhanced metabolic phenotype highlighted by a significant increase in both basal and maximal respiratory capacity [8]. The dynamic interplay between glycolysis and oxidative metabolism was evaluated in TamR/TetOn-V and TamR/TetOn-AAA cells by analyzing both the oxygen consumption rate (OCR) and the extracellular acidification rate (ECAR) after 48 and 72 h of Dox stimulation. Interestingly, Dox-induced FoxO3a overexpression caused a shift in the metabolic potential of TamR BCCs to a low efficient metabolic phenotype (Figure 2A and Figure 3A). The effect was emphasized after 72 h of Dox stimulation (Figure 2C and Figure 3C).

As shown in Figure 2B,D, a significant reduction in ATP-coupled respiration, leaked respiration (proton leak) and maximal respiration was observed in TamR/TetOn-AAA in response to the sequential addition of oligomycin, FCCP, Rotenone and antimycin A to the cells. These results suggest that the electron transport chain activity is affected by FoxO3a expression. In addition, a significant reduction in glycolysis, glycolytic capacity and glycolytic reserve was observed in TamR/TetOn-AAA with respect to TamR/TetOn-V in response to the sequential injections of glucose, oligomycin and 2-deoxyglucose. As for OCR, the effect was emphasized by the increased expression of FoxO3a (Figure 3A–D). Interestingly, the expression of FoxO3a in TamR BCCs caused a regression from a high energetic phenotype to a quiescent cell phenotype, indicated by the ratio between OCR and ECAR (Figure 3E,F). Considering that the energy deriving from an increased glycolysis supports the proliferation of cancer cells, FoxO3a inhibitory effect on OCR and on ECAR shows that its protective role in BCCs also occurs through the reduction in the ATP production.

**Figure A1:**
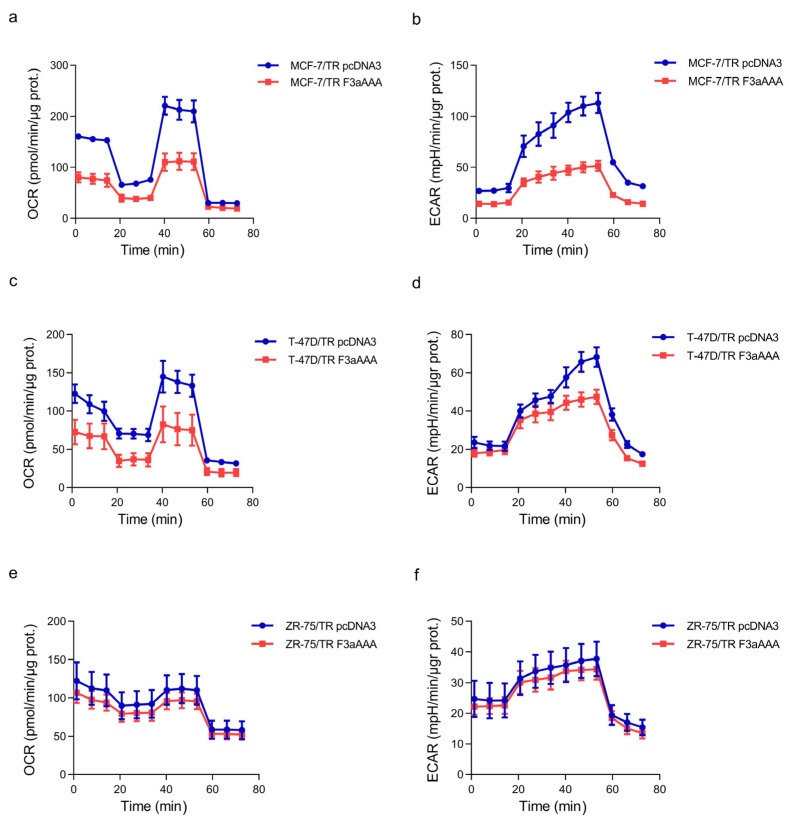
Effect of FoxO3a on OCR and on ECAR in other TamR BCC lines. TamR cells were transfected in suspension with F3aAAA or pcDNA3.1 plasmids. After 72 h, OCR and ECAR were evaluated in MCF-7/TR (**a**,**b**), T-47D/TR (**c**,**d**), and ZR-75/TR (**e**,**f**). OCR was measured under basal conditions followed by the sequential addition of 10 µM Oligo-mycin, 9 µM FCCP, 10 µM Rotenone and 10 µM Antimycin A. ECAR was measured under basal conditions followed by the sequential injection of 80 mM glucose, 9 µM oligomycin, and 500 mM 2-deoxyglucose. Each data point represents an OCR and ECAR measurements. Representative line graphs of three independent experiments, normalized vs. protein content, are shown.

**Figure A2:**
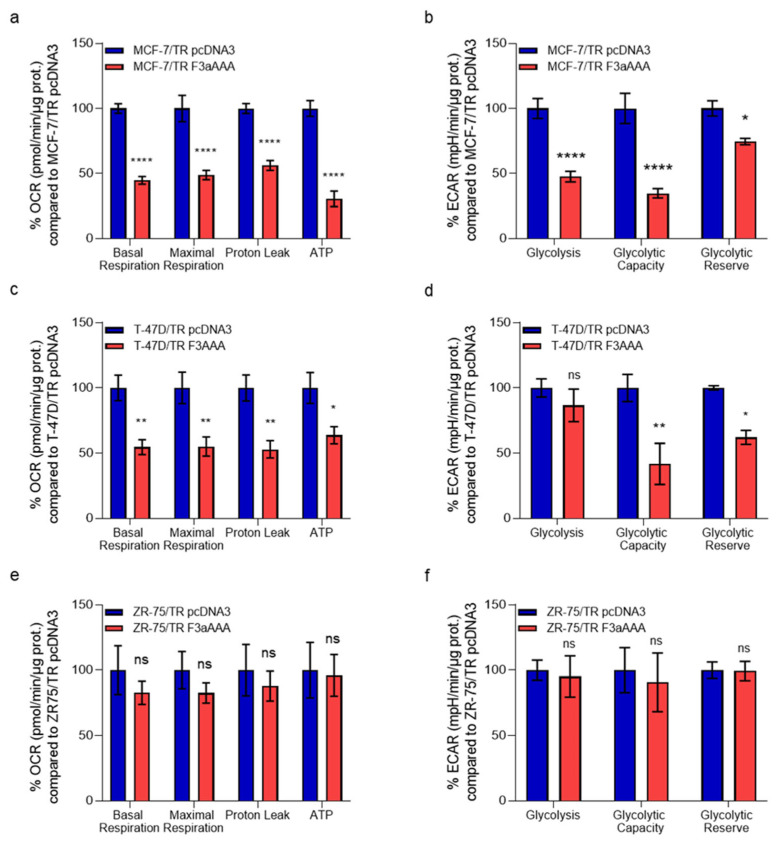
Effect of FoxO3a on OCR and on ECAR in other TamR BCC lines. TamR cells were transfected in suspension with F3aAAA or pcDNA3.1 plasmids. After 72 h, OCR and ECAR were evaluated in MCF-7/TR (**a**,**b**), T-47D/TR (**c**,**d**), and ZR-75/TR (**e**,**f**). OCR was measured under basal conditions followed by the sequential addition of 10 µM Oligo-mycin, 9 µM FCCP, 10 µM Rotenone and 10 µM Antimycin A. Histograms represent the % of basal and maximal respiration, proton leak and ATP calculated at 72 h, compared to pcDNA3.1 cells. ECAR was measured under basal conditions followed by the sequential injection of 80 mM glucose, 9 µM oligomycin, and 500 mM 2-deoxyglucose. Histograms represent the % of glycolysis, glycolytic capacity, glycolytic reserve calculated at 72 h, compared to pcDNA3.1 cells. Statistical significances are calculated by using 2way ANOVA Šídák’s multiple comparisons test, evaluating the differences between F3aAAA and pcDNA3 samples (ns = not significant, * *p* < 0.05, ** *p* < 0.005, **** *p* < 0.00005).

ECAR and OCR results have been confirmed in both MCF-7/TR (Figure A1a,b and Figure A2a,b) and T-47D/TR (Figure A1c,d and Figure A2c,d) BCCs transiently transfected with F3aAAA. On the other hand, ZR-75/TR only showed a trend toward a reduction in both glucose and oxygen consumption following FoxO3a over-expression (Figure A1e,f and Figure A2e,f). 

### 3.3. FoxO3a Reduces Glycolysis Efficiency and LDH Activity in TamR BCCs

**Figure 4:**
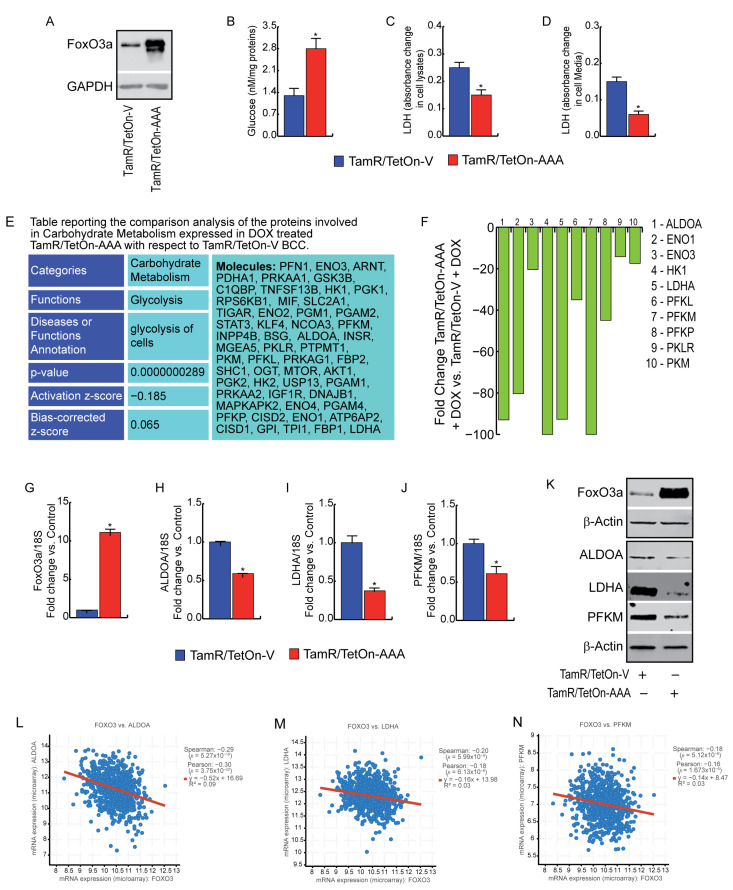
Influence of FoxO3a expression on glycolysis of TamR breast cancer cells. TamR/TetOn-AAA and TamR/TetOn-V were plated, left to attach and then treated with Dox 1 µg/mL for 48 h to induce FoxO3a expression. (**A**) FoxO3a overexpression was determined by WB. GAPDH = loading control. Glucose content (**B**) LDH (Lactate dehydrogenase) activity in cell lysates (**C**) and in the growing media (**D**) were analyzed as described in Section 2. Data are reported as the mean ± SD of three independent experiments (at least in triplicate). Statistical significances are calculated by using Student’s *t*-test evaluating the differences between TamR/TetOn-AAA and TamR/TetOn-V samples (* *p* < 0.05). Proteins involved in Carbohydrate Metabolism differentially expressed in the comparison analysis in TamR/TetOn-AAA with respect to TamR/TetOn-V (**E**). The expression fold changes of reported proteins were evaluated in TamR/TetOn-AAA with respect to TamR/TetOn-V by using Ingenuity Pathway Analysis (Qiagen), fixing the cut-off to 1.6 (**F**). Proteomic data were confirmed by RNA and WB analysis. To this aim, a duplicate set of cells was treated with Dox 1 µg/mL for 48 h. The RNA (**G**–**J**) and protein (**K**) expressions of FoxO3a, AldoA, LDHA and PFKM were assessed. mRNA content was normalized vs. the relative 18S rRNA content, and β-actin was employed as a loading control. (**L**–**N**) Analysis of genes involved in carbohydrate metabolism that correlated with FOXO3 (cBioPortal dataset) [26], Spearman and Pearson correlation coefficient with the respective *p*-value are reported.

To give more insight into the effect of FoxO3a on the glycolytic pathway, we used a combination of metabolic phenotyping and proteomics analysis. First, we analyzed the cellular glucose content in our cell lines over-expressing or not FoxO3a (Figure 4A), evidencing an accumulation of glucose in TamR/TetOn-AAA with respect to TamR/TetOn-V (Figure 4B). Glucose storage could be caused by reduced LDH activity in TamR/TetOn-AAA cells, as detected in both protein lysates (Figure 4C) and cell incubation media (Figure 4D). 

Proteomic analysis confirmed that F3a-AAA over-expression strongly affects carbohydrate metabolism, reducing the efficacy of glycolysis through a significant decrease in relevant enzymes of the canonical pathways involved in carbohydrate metabolism compared to the control (TamR/TetOn-V) cells (*p* = 2.89–8) (Figure 4E), some of which are reported in Figure 4F. In particular, we observed that the expressions of Aldolase A (ALDOA), LDHA and phosphofructokinase (PFK) are affected by F3a-AAA at both the mRNA (Figure 4G–J) and protein level (Figure 4K), suggesting a potential involvement of FoxO3a in the transcriptional regulation of these enzymes.

**Figure A3:**
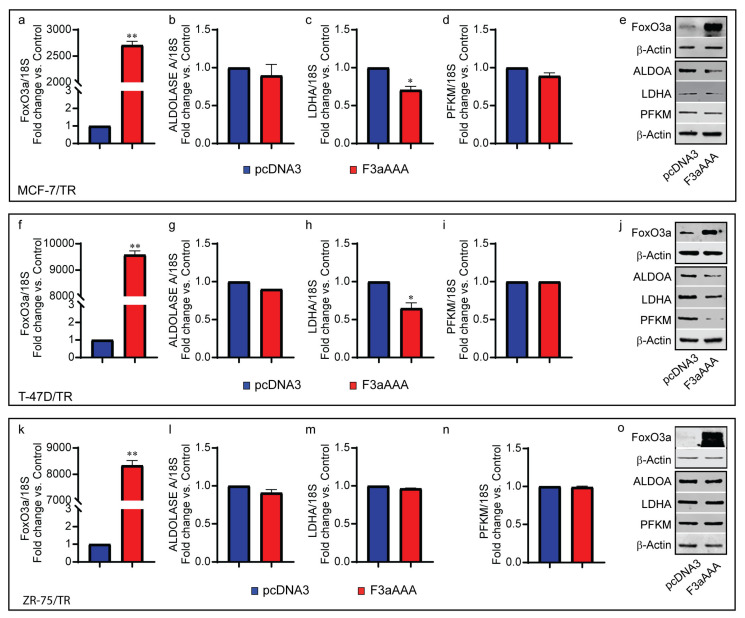
Effect of FoxO3a on relevant enzymes involved in carbohydrate metabolism. A double set of cells was transiently transfected in suspension with F3aAAA and pcDNA3 plasmids for 48 h. FoxO3a, PFKP, LDHA, and Aldolase A expressions were assessed at the RNA and protein level in Tam-resistant MCF-7/TR (**a**–**e**), T47D/TR (**f**–**j**) and ZR-75/TR (**k**–**o**). mRNA content was normalized vs. the relative 18S rRNA content, and β-actin was employed for loading controls (* *p* < 0.05, ** *p* < 0.005).

FoxO3a strongly reduced ALDOA, LDHA and PFK protein levels also in transiently transfected MCF-7/TR (Figure A3a–e) and T-47D/TR (Figure A3f–j), but only LDHA RNA transcripts were affected by F3aAAA overexpression at the considered time point. On the other hand, as expected from seahorse results, FoxO3a induction did not lead to any change in both the RNA and protein expression of these enzymes in ZR-75/TR (Figure A3k–o). Despite this inconsistency, we found that FoxO3a RNA expression resulted inversely correlated to those of ALDOA (Figure 4L), LDHA (Figure 4M) and PFK (Figure 4N) in a cBioPortal dataset [26] (see Section 2). These data suggest that, with only a few exceptions, FoxO3a’s inverse correlation with enzymes of the canonical carbohydrate metabolism might be considered a general phenomenon in BC patients.

### 3.4. FoxO3a Increases Mitochondrial Biogenesis and Reduces Mitochondrial Functionality, Increasing ROS Production in TamR BCCs

**Figure 5:**
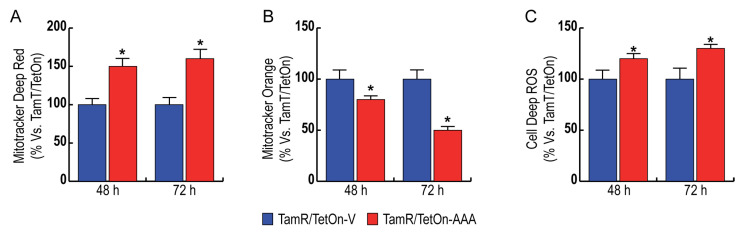
Impact of Nuclear FoxO3a on mitochondrial biogenesis, mitochondrial functionality, and ROS production. TamR/TetOn-AAA and TamR/TetOn-Vcells were serum starved for 24 h, treated with Dox 1 µg/mL for the indicated time points and to subjected to FACS analysis to evaluate: mitochondrial mass (**A**) using MitoTracker Deep-Red dye; mitochondrial membrane potential (**B**) using MitoTracker Orange dye; ROS production (**C**) using CellROX Deep Red Reagent. Data are the mean ± SD of three independent experiments (at least in triplicate) reported as % of variation with respect to vector (* *p* < 0.01).

TamR BCCs have been previously described as being characterized by increased mitochondrial function and biogenesis [8]. Considering that F3a expression alters several metabolic functions involving mitochondria, we deepened our findings on these organelles. Particularly, the expression of nuclear F3a in TamR BCC caused a significant increase in the mitochondrial population (about 50%) of TamR BCC (Figure 5A). An additional slight increase in mitochondrial biogenesis was observed after 72 h of Dox treatment (Figure 5A). Interestingly, the increase in mitochondrial mass was not paralleled by an increase in mitochondrial membrane potential (Figure 5B), resulting in significantly reduced TamR/TetOn-AAA cells (−25% after 48 h and −50% after 72 h of Dox treatment). The reduced mitochondrial functionality was paralleled by an elevated formation of reactive oxygen species (ROS) (Figure 5C), indicating a perturbation in the electron transport. 

### 3.5. FoxO3a Impairs NADPH Production through the Pentose Phosphate Pathway (PPP) in TamR BCCs

**Figure 6:**
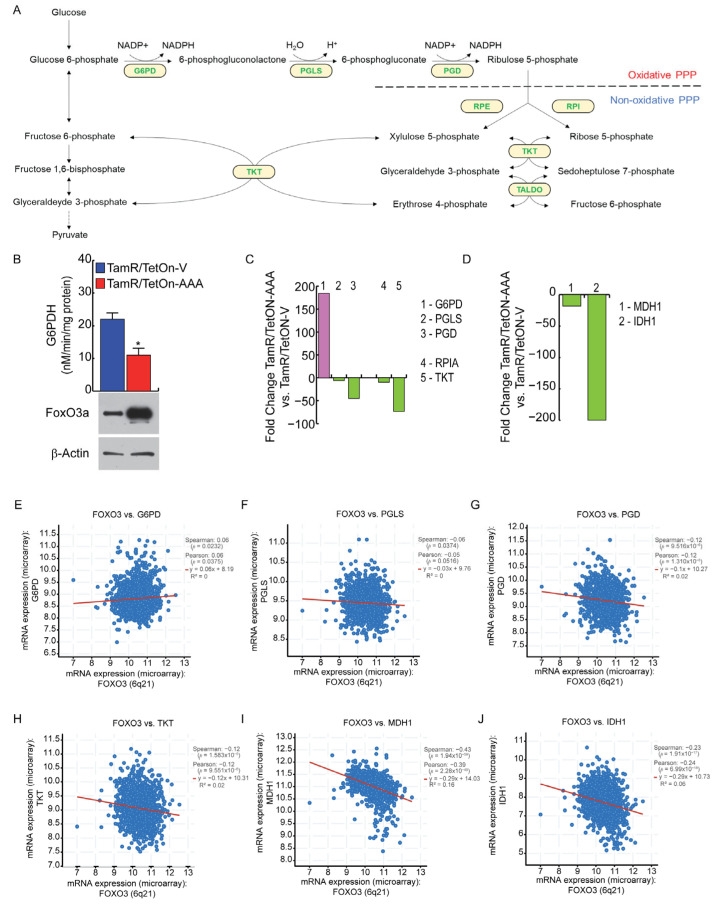
FoxO3a affects the Pentose Phosphate Pathway (PPP) in TamR BCCs. Cells were treated as shown in Figure 4. (**A**) Schematic representation of the PPP, composed of an oxidative and a non-oxidative arm. Under high oxidative stress conditions, metabolites from the PPP non-oxidative arm re-enter into glycolysis to refill the oxidative arm for the synthesis of NAPDH. (**B**) G6PDH activity was assessed as described in Section 2. Data are reported as the mean ± SD of three independent experiments. Statistical significances are calculated by using Student’s *t*-test (* *p* < 0.05). FoxO3a overexpression was determined by WB; β-actin was used as loading control. (**C**) The expression fold changes of proteins of the oxidative and non-oxidative branches of the PPP were evaluated; cut off to 1.5-fold-change in protein levels, with *p* < 0.05, was considered significant. (**D**) The expression fold changes of indicated proteins were evaluated as in (**C**). (**E**–**J**) Analysis of genes involved in NADPH production that correlated with FOXO3 (cBioPortal). Spearman and Pearson correlation coefficients with the respective *p*-values are reported.

Since glycolytic inhibition generally promotes NADPH formation, we also evaluated the effect of FoxO3a overexpression on the main route for cellular NADPH production, the pentose phosphate pathway (PPP), which is composed of the oxidative and non-oxidative arms (Figure 6A). The key rate-limiting enzyme of the PPP oxidative arm is the glucose-6-phosphate dehydrogenase (G6PDH), which has been reported to be activated or overexpressed in several cancers [27]. Based on this evidence, we expectedly found that G6PDH activity was reduced by about half in TamR/TetOn-AAA cells with respect to TamR/TetOn-V (Figure 6B). Nevertheless, proteomic analysis revealed that the reduced G6PDH activity was not due to a reduction in enzyme expression, which, instead, resulted in a strong increase in TamR/TetOn-AAA compared to the control cells (Figure 6C), rather suggesting a possible reduction in substrate availability. However, the protein levels of the other two enzymes of the oxidative arm of PPP, 6-phosphogluconolactonase (PGLS) and 6-phosphogluconate dehydrogenase (PGD), which were reproducibly decreased in TamR/TetOn-AAA cells (Figure 6C). As for G6PDH, PGD also mediates a reaction that generates NADPH and its decreased expression confirms that NADPH generation through the oxidative PPP is impaired in TamR/TetOn-AAA. These results suggest that the ROS-mediated redirection of glucose into PPP is less effective in FoxO3a-overexpressing TamR BCCs. 

Several enzymes in the PPP non-oxidative arm, including Ribose 5-Phosphate Isomerase A (RPIA) [28] and Transketolase (TKT) [29,30] have been found to be involved in cancer. Particularly, TKT holds a key position, since it connects PPP with glycolysis, affecting the production of the antioxidant NADPH, and its blockade has been reported to increase oxidative stress, making tumor cells more vulnerable to therapeutic treatment [29]. Accordingly, TKT expression was dramatically reduced in our TamR/TetOn-AAA cell model (Figure 6C). 

Other cytosolic enzymes, including Malate dehydrogenase 1 (MDH1) and Isocitrate Dehydrogenase 1 (IDH1), are also involved in the production of NADPH. The inhibition of MDH1, a key enzyme in the glutamine catabolic pathway, has been recently reported to sensitize cancer cells to oxidative stress, decreasing cell proliferation and inhibiting tumor growth in vivo [31], and its targeting is being suggested as a therapeutic approach [32]. Similarly, IDH1 has been related to tumor development, and changes in IDH1 expression levels or gene mutations have been reported in several tumors [33,34]. Our proteomic data reveal a decreased expression of both MDH1 and IDH1 in TamR/TetOn-AAA cells (Figure 6D), suggesting the breakdown of NADPH cytosolic production driven by FoxO3a. 

Proteomic data were confirmed by analyzing clinical data from BC patients’ cohorts, which showed that FoxO3a expression correlates directly to G6PDH (Figure 6E) and inversely to PGLS (Figure 6F), PGD (Figure 6G), TKT (Figure 6H), MDH1 (Figure 6I) and IDH1 (Figure 6J).

## 4. Discussion

Recently, FoxO3a has been recognized as a promising therapeutic target in cancers and enhancing its expression appears to be relevant to tumor treatment [35]. Acquired Tam resistance is the major limitation in the efficacy of Tam in ~50% of ER+ BCs; therefore, overcoming this drawback with novel therapeutic strategies is highly needed. MCF-7 cells are sensitive to Tam and display a prevalently glycolytic metabolic phenotype. Conversely, high mitochondrial activity and low glucose uptake were observed in Tam resistance [8]. FoxOs transcription factors have been involved in the control of cellular energy metabolism [36]; therefore, we hypothesized a role of FoxO3a in the disruption of the TamR BC metabolic phenotype and in restoring the sensibility to Tam. Herein, by using multifaceted approaches, such as proteomics analysis and metabolic phenotyping, we discovered a novel protective role of FoxO3a in human BC since it interferes with various features of tumor metabolism, enabling TamR BCCs to reacquire sensibility to Tam. These results perfectly fit with our recently published studies [19], showing that the expression of an active FoxO3a in TamR BCCs (TamR/TetOn-AAA) re-established the response to Tam, causing a strong reduction in cell proliferation compared to control TamR BCCs (TamR/TetOn-V). The characterization of the metabolic phenotype of TamR/TetOn-AAA versus TamR/TetOn-V cells was first evaluated through the use of the Seahorse XF96 Analyzer to determine the dynamic interplay between glycolysis and oxidative metabolism. Intriguingly, FoxO3a induction reduced metabolic flux, with a significant decrease both in glycolytic rate (ECAR) and in oxidative mitochondrial metabolism (OCR). These results are consistent with the previously reported data, which suggests that FoxO3a is an important determinant in restraining oncogenic glycolysis in cancers since its knockdown is sufficient to activate cellular glycolysis and increase cell resistance to apoptosis [14]. The effect of FoxO3a on ECAR and on OCR was also confirmed in transiently transfected MCF-7/TR and T-47D/TR cell lines but not in ZR-75/TR, which showed only a slight, not significant, decrease in both glucose and oxygen consumption. These data are in line with our previously published results, showing that FoxO3a overexpression was able to reduce the migrating and invasive potential of MCF-7/TR and T-47D/TR cell lines but not that of ZR-75/TR [21]. In addition, salient metabolic differences among ZR-75 (more phosphorylative) and MCF-7 (highly glycolytic) cells have been reported, and the use of specific genes and energy pathways probably impart more aggressiveness to ZR-75 compared to MCF-7 [37]. Nonetheless, such different behavior might also depend on the fact that, different from MCF-7 and T-47D, ZR-75 are PTEN-mutant cells [38], showing basal hyperactive AKT signaling [39], further emphasized by the condition of Tam resistance, which results, in turn, in very low levels of FoxO3a [19]. In this scenario, FoxO3a transient expression might not be sufficient to counteract such a sustained PI3K/AKT pathway. 

Therefore, since the contribution ratio of glycolysis versus OXPHOS for the total ATP yield varies in different cell systems, growth states and microenvironments, we decided to delve comprehensively into the metabolic status of TamR/TetOn-AAA cells, scanning several biochemical pathways. The study was conducted through the use of both biochemical and WB assays as well as by using proteomics analysis for each metabolic branch considered. By approaching the glycolytic machinery, we observed an increase in the glucose content and a reduction in PFK1 (PFKM) expression in TamR/TetOn-AAA cells. In addition, LDH-A expression and the lactate production both inside and outside the cells decreased. LDH-A is a vital metabolic enzyme that is associated with cancer development, and it catalyzes the forward and backward conversion of pyruvate to lactate. Elevated LDH has been recognized as a poor prognostic indicator in breast cancer [40] and the inhibition of LDH-A has an anti-proliferative effect on breast tumors [41]. Consistently, our results show a significant reduction in LDH expression and activity in TamR/TetOn-AAA cells, which might be a consequence of PFK1 decrease, resulting in the accumulation of fructose-6-phosphate, which is then isomerized to glucose-6-phosphate and, in turn, is diverted into the pentose phosphate pathway (PPP). Indeed, the lower glycolytic efficiency observed in TamR/TetOn-AAA cells could also be due to the reduced activity of G6PDH, the rate-limiting catalyzing enzyme of the PPP and the main cellular source of NADPH. Therefore, FoxO3a seems to decrease the glucose catabolism in TamR/TetOn-AAA cells by lowering both the glycolysis and the PPP rates. These results may explain, at least in part, the glucose accumulation found in TamR/TetOn-AAA cells. Rapidly proliferating cancer cells constantly demand nucleotides and materials for biosynthesis, and the PPP is frequently upregulated in many tumors, accounting for approximately 60% of NADPH production in humans. Other than the PPP, MDH1 and IDH1 cytosolic enzymes are also involved in the production of NADPH. The proteomics analysis indicated the decreased expression of both MDH1 and IDH1 (Figure 6), suggesting a breakdown of NADPH cytosolic production driven by FoxO3a. Among the multiple mechanisms that have been proposed for the development of Tam resistance, Lisanti et al. hypothesized that it occurs through a metabolic shift in MCF-7 cells from a glycolytic to an oxidative state and that restoring their glycolytic state may overcome Tam resistance [8]. Since the metabolic phenotype of MCF-7 TamR cells is prevalently phosphorylative, it is clear that mitochondria play a central role in the acquisition of resistance to Tam. Indeed, both tricarboxylic acid (TCA) and electron transport chain (ETC) are highly functional in TamR BCCs [8]. 

Notably, FoxO3a strongly reduced mitochondrial functionality in TamR/TetOn-AAA with respect to the control cells, attenuating the hyper-phosphorylative phenotype of TamR cells, and the concomitant ROS increase observed is consistent with a perturbation in electron transport [42]. Our data well fit with a previously published study showing that FoxO3a activation reduces the mitochondrial DNA copy number as well as the expression of respiratory complexes and mitochondrial proteins, leading to an overall inhibition of mitochondrial respiratory activity [16]. Intriguingly, the decrease in the mitochondrial membrane potential was rather paralleled by an increase in mitochondrial biogenesis. Although the effect might be explained as a compensatory response of the cell to mitochondrial dysfunction, it has to be underlined that FoxO3a is a direct transcriptional regulator of peroxisome proliferator-activated receptor γ coactivator 1α (PGC-1α) [43], which has emerged as a master regulator of mitochondrial biogenesis [44]. Therefore, the potential implications of the FoxO3a/PGC-1α axis in the reversion of Tam resistance need further investigations. 

## 5. Conclusions

Here, we report, for the first time, an additional mechanism through which an active FoxO3a can counteract Tam resistance in BCCs. Our data demonstrate how FoxO3a can affect multiple biochemical pathways of BC cell metabolism, spanning from the impairment of glucose breakdown, mitochondrial functionality and NADPH production to the induction of ROS production. These FoxO3a-induced metabolic perturbations might explain, at least in part, the restoration of the response to Tam treatment that we recently described in FoxO3a overexpressing TamR cells [19]. Ongoing studies in our laboratory are aimed at elucidating the molecular mechanisms governing each metabolic pathway (i.e., glycolysis, mitochondrial respiration and PPP) affected by FoxO3a. In addition, the impact on the metabolism of drugs that are able to enhance FoxO3a activity (e.g.**,** AKT inhibitors [25] or lamotrigine [20]) will be investigated as well. The results derived from these efforts will emphasize the idea that anti-cancer therapies exploiting FoxO3a might be beneficial to those BC patients who have developed resistance to Tam treatment. 

## Data Availability

Data are contained within the article and Supplementary Materials.

## Supplementary Materials

The following supporting information can be downloaded at: https://www.mdpi.com/article/10.3390/cells12242777/s1, Table S1: List of the primer sequences used in the study. Figure S1. (A) MCF-7 cells were transfected in suspension with F3aAAA and pcDNA3 plasmids. After 6 h, cells were serum-starved for 16 h and then shifted to 5% PRF-CT +/− 4-OHT (1 μM). After 24 h, 48 h and 72 h cells were then harvested by trypsinization and counted using trypan blue dye exclusion assay. (B) TamR/TetOn-V and TamR/TetOn-AAA cells were serumstarved for 16 h and then switched to 5% PRF-CT plus 1 μM 4-OHT and treated or not with Dox 1 μg/mL. After 24 h, 48 h and 72 h cells were detached from the culture plate and subjected to cell counting in trypan blue dye exclusion assay. Statistical significances are calculated by using One Way Anova (* *p* < 0.05).
