# Supplementary material for: FoxO3a Drives the Metabolic Reprogramming in Tamoxifen-Resistant Breast Cancer Cells Restoring Tamoxifen Sensitivity
Source: Cells. 2023 Dec 6;12(24):2777. doi: 10.3390/cells12242777 (PMC10742319; doi:10.3390/cells12242777)
Supplement: Supplementary file 1 [file cells-12-02777-s001.zip › cells-2657395-supplementary.pdf]

Supplementary figure S1

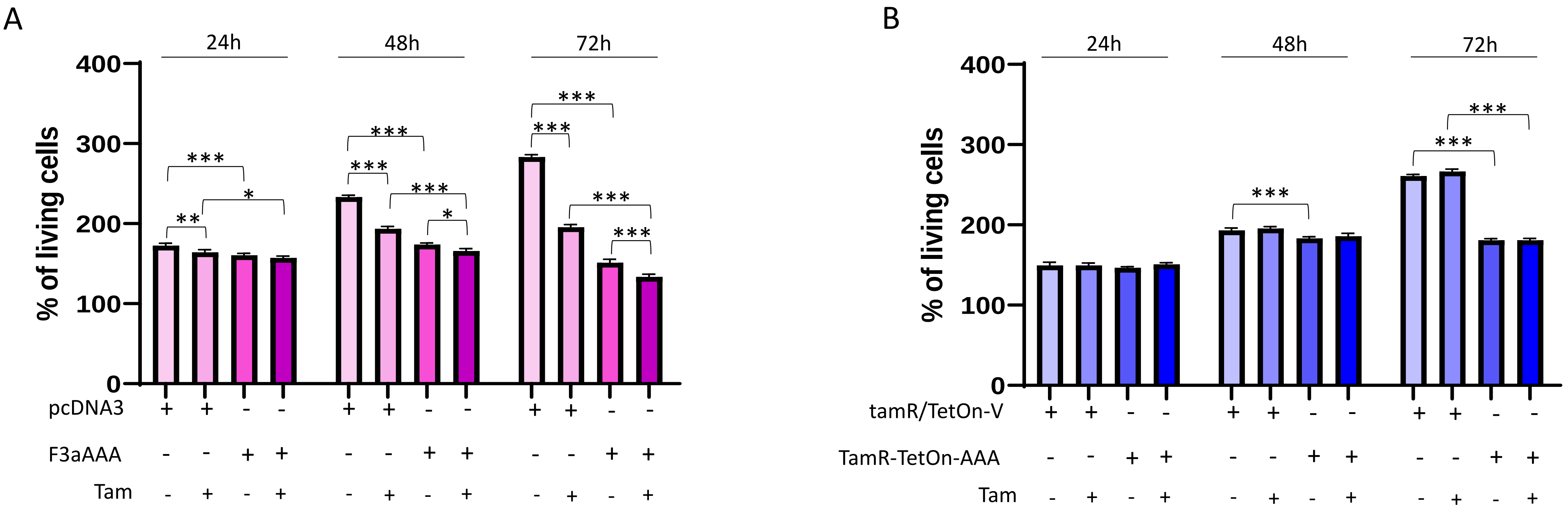

Figure S1. (A) MCF-7 cells were transfected in suspension with F3aAAA and pcDNA3 plasmids. After 6 h, cells were serum-starved for 16 h and then shifted to 5% PRF-CT +/− 4-OHT (1 µM). After 24 h, 48 h and 72 h cells were then harvested by trypsinization and counted using trypan blue dye exclusion assay. (B) TamR/TetOn-V and TamR/TetOn-AAA cells were serum-starved for 16 h and then switched to 5% PRF-CT plus 1 µM 4-OHT and treated or not with Dox 1 µg/mL. After 24h, 48h and 72h cells were detached from the culture plate and subjected to cell counting in trypan blue dye exclusion assay. Statistical significances are calculated by using One Way Anova (* $p<0.05$).

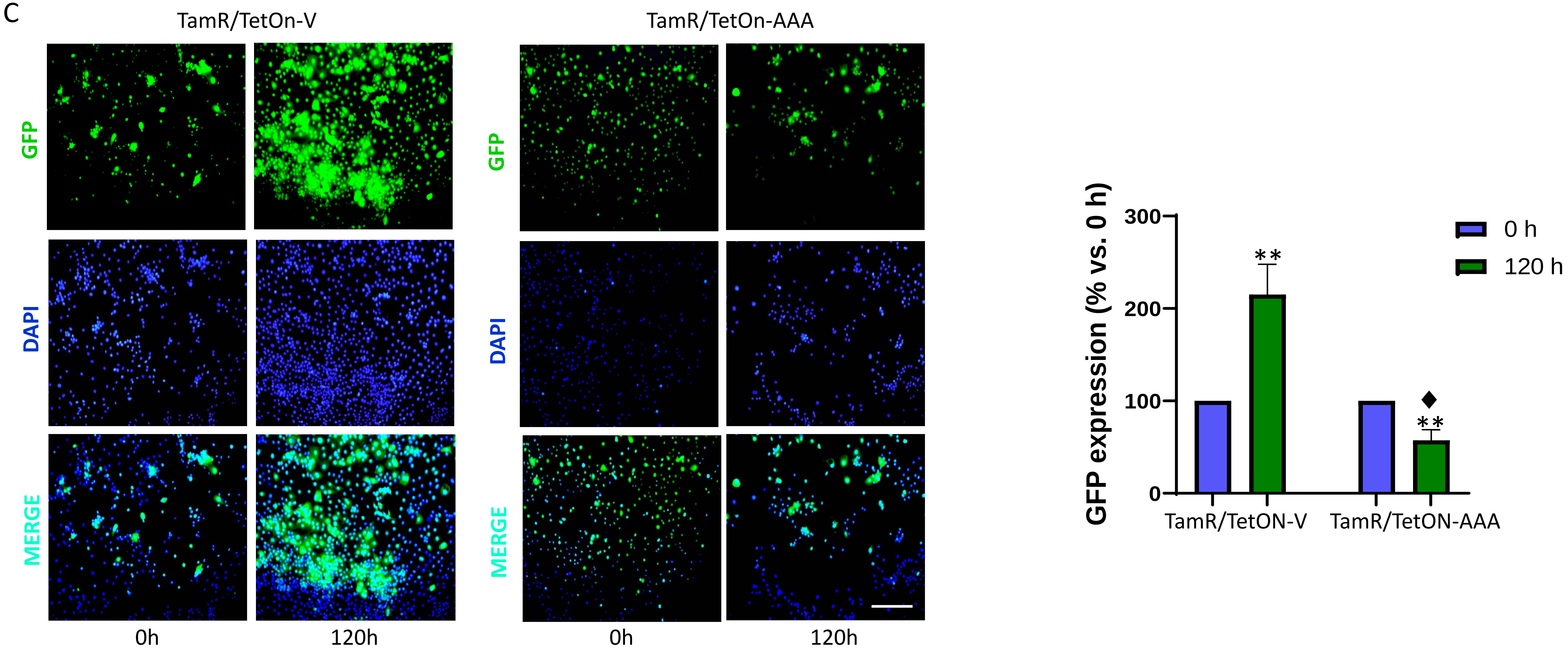

Figure S1 (cont). (C) GFP expressing cells (green) were photographed under a fluorescent microscope at 10x magnification at time 0 and after 120 hours of Dox (1µg/ml) treatment. DAPI staining (blue) was used for nuclei detection. Merged images of the GFP and DAPI signals are also reported. Scale bar = 100 µm. GFP expressing cells and nuclei were counted by means of ImageJ and reported in the graph on the right. Data are the mean ± SD of three independent experiments, reported as variation (%) *vs* time 0 (** p<0.01) or *vs* the correspondent time point in TamR/TetOn cells (◆ p<0.001).

Table S1. List of primers used in the study for RT-PCR assays.

**FoxO3a**	CGCACAAACGGCTCACTCTG	GGGGGCTTTTCCGCTCTTC
**18S**	CGGCGACGACCCATTCGAAC	GAATCGAACCCTGATTCCCCGTC
**LDHA**	ATCTTGACCTACGTGGCTTGGA	CCATACAGGCACACTGGAATCTC
**PFKP**	GCATGGGTATCTACGTGGGG	CTCTGCGATGTTTGAGCCTC
**ALDOLASE A**	GACACTCTACCAGAAGGCGGAT	GGTGGTAGTCTCGCCATTTGTC

Figure 1 (C)

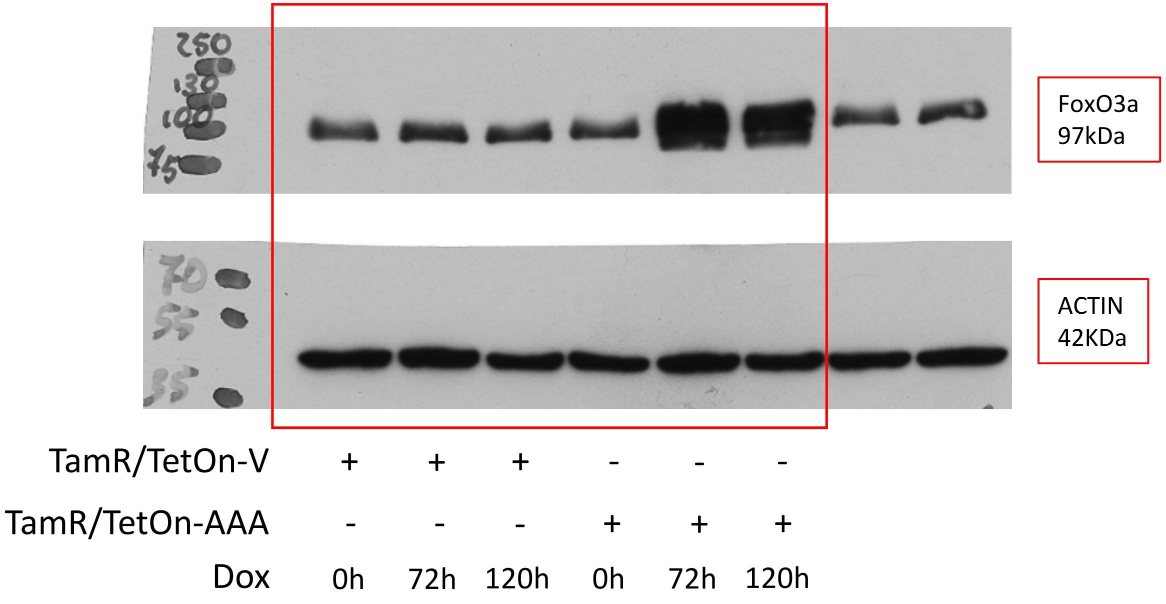

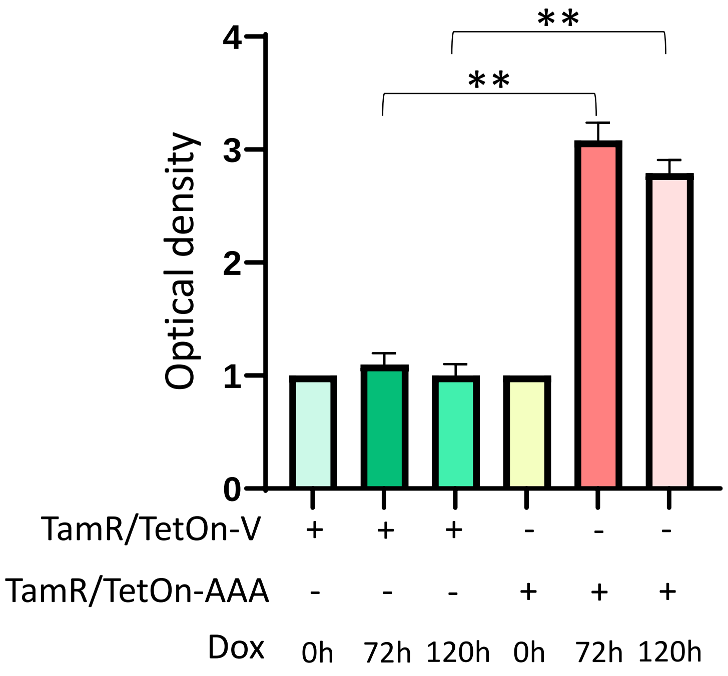

Lane	1	2	3	4	5	6
FoxO3a	12.471.374	14.296.598	12.138.142	12.573.934	40.887.692	36.801.391
Actin	20.123.045	20.397.096	16.828.111	15.810.042	17.439.357	16.739.207
Ratio	0,6170723	0,7009134	0,7256134	0,7953131	2,3445641	2,1985146
Normalized ratio	1	1.1	1	1	2,9	2,7

Figure 2 (E)

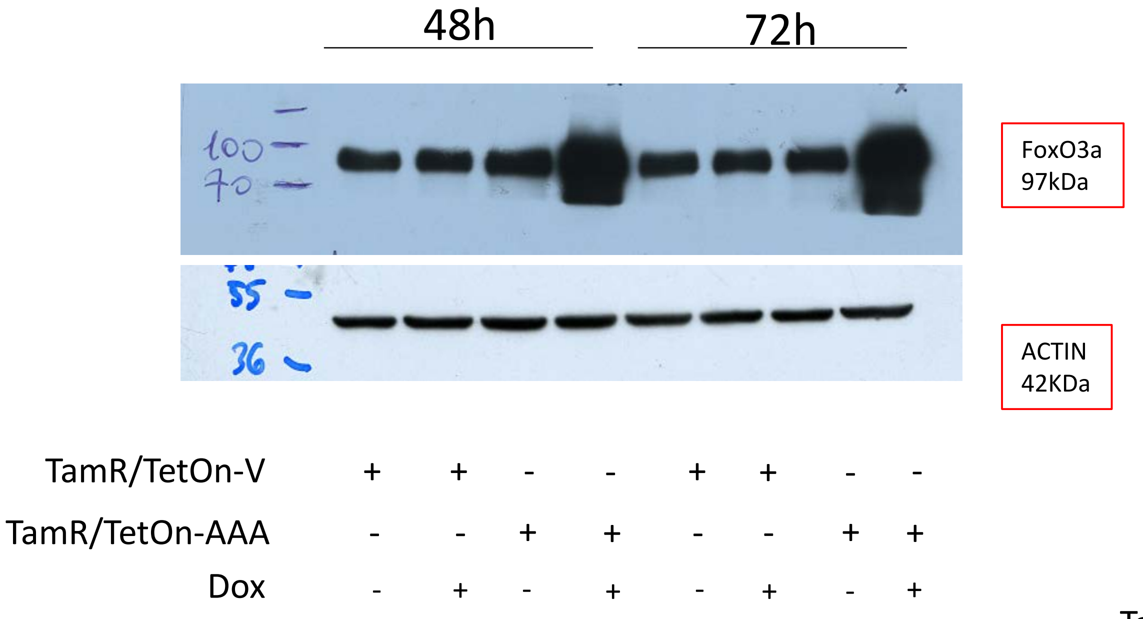

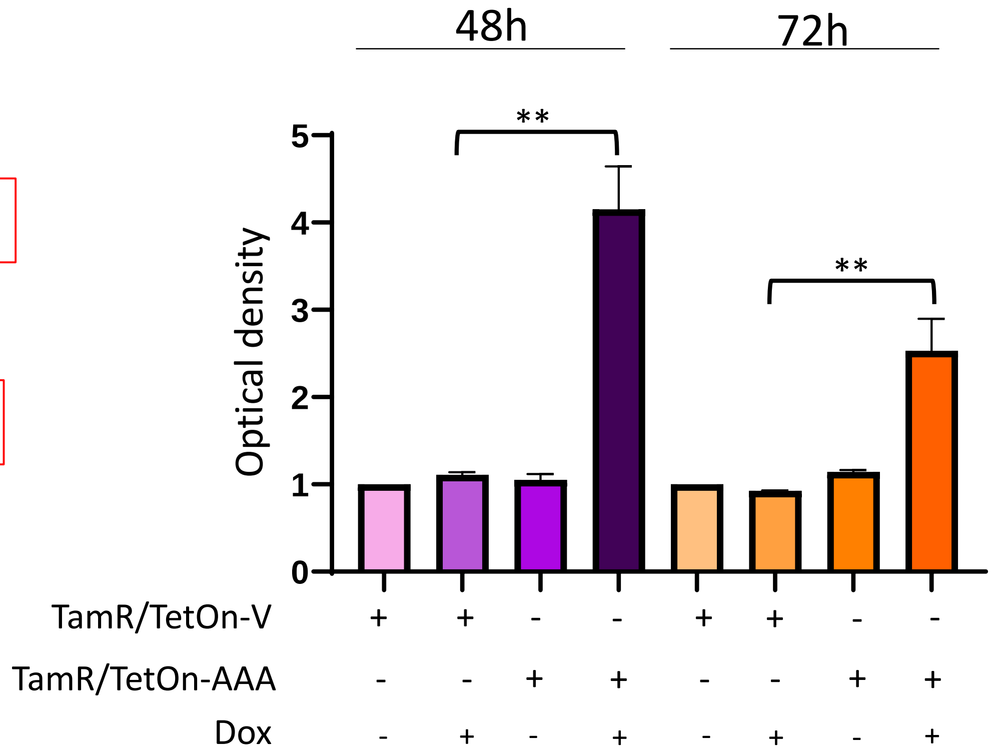

Lane	1	2	3	4	5	6	7	8
FoxO3a	17.726.628	17.799.381	18.547.037	67.980.222	19.344.712	17.346.811	20.411.522	47.309.971
Actin	16.895.386	16.906.270	16.812.189	15.929.178	14.125.056	14.613.670	14.457.298	14.685.422
Ratio	1,0491993	1,0528272	1,1031898	4,2676541	1,2789844	1,1870263	1,411849	3,2215601
Normalized ratio	1	1	1	4,1	1	0,9	1,1	2,5

Figure 4 (A)

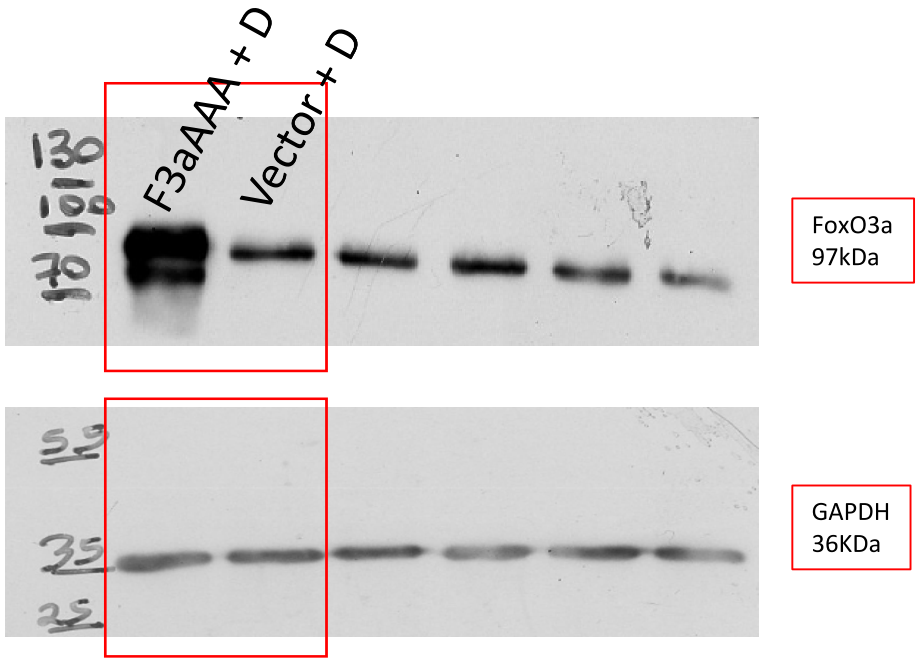

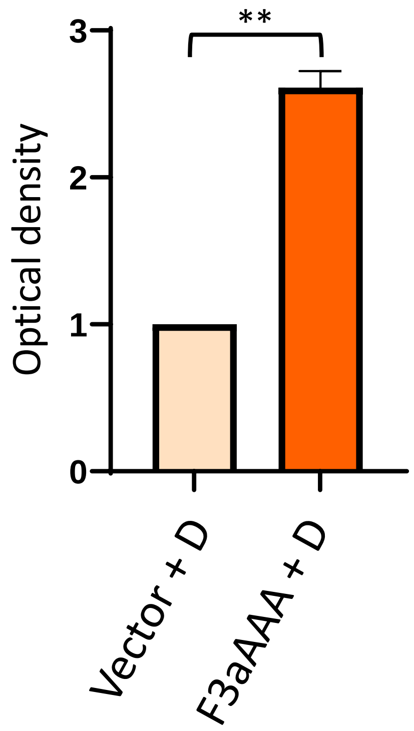

Lane	1	2
FoxO3a	29.799.399	10.276.187
GAPDH	14.068.296	12.619.417
Ratio	2,1181953	0,8143155
Normalized ratio	2,6	1

Figure 4 (K)

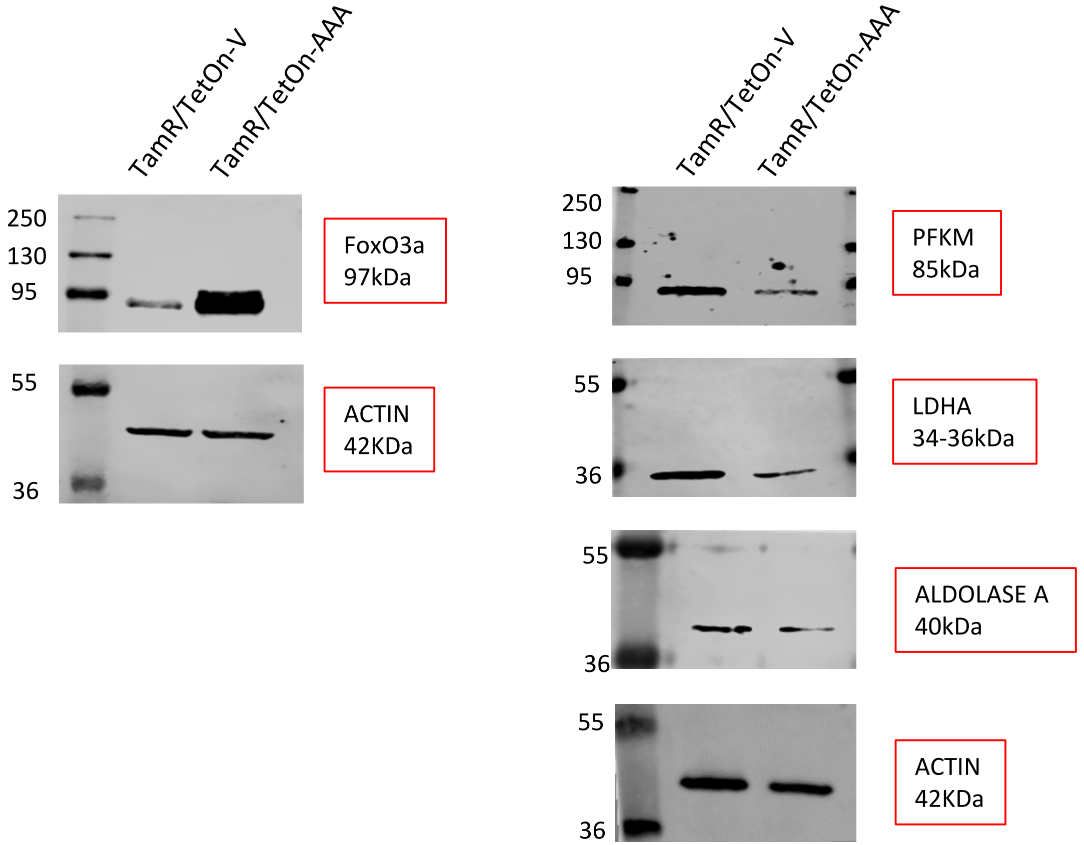

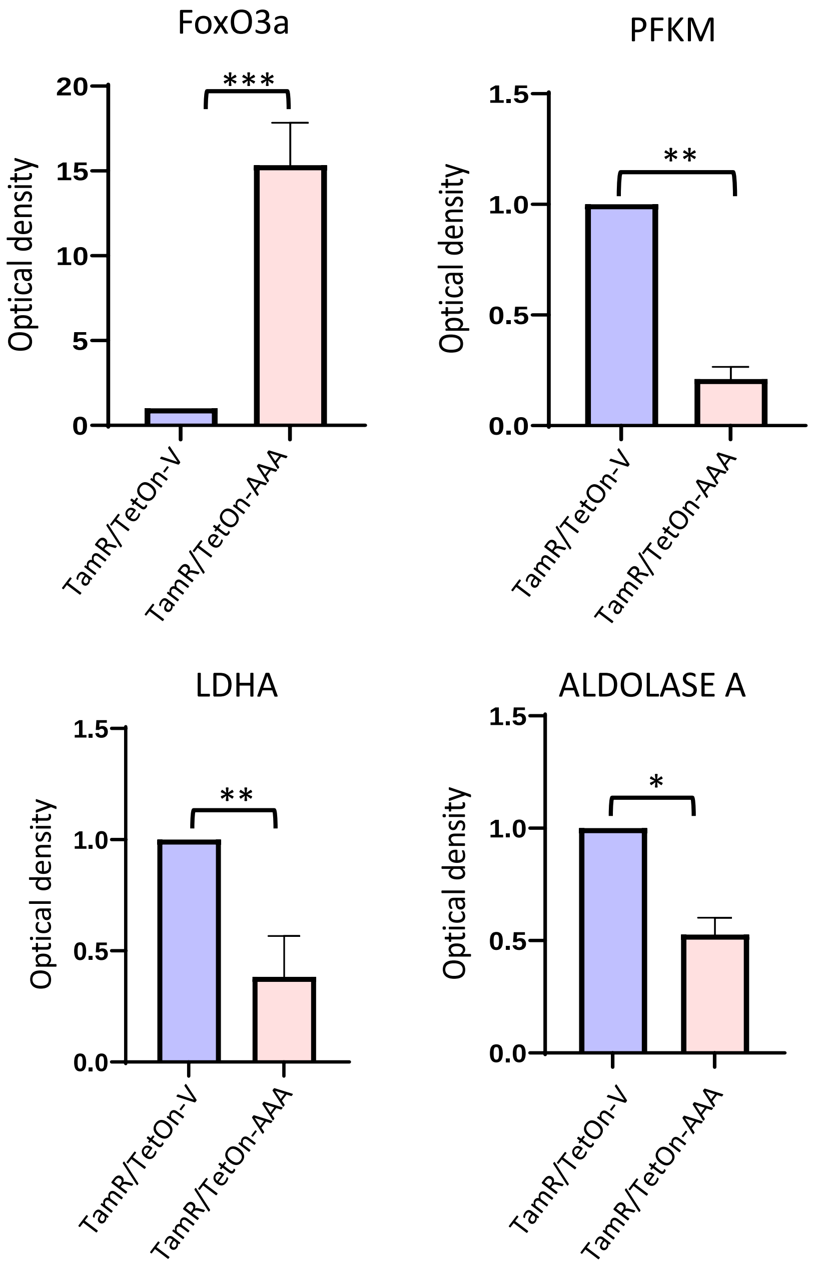

Lane	1	2
FoxO3a	0,23	3,2
Actin	49,45	46,96
Ratio	0.004651	0,0682302
Normalized ratio	1	15

Lane	1	2
LDHA	0,705	0,359
Actin	2,966	3,563
Ratio	0.237693	0,1007577
Normalized ratio	1	0,4

Lane	1	2
PFKM	0,126	0,0189
Actin	3,189	3,795
Ratio	0.039510	0,0052437
Normalized ratio	1	0,2

Lane	1	2
ALDOLASE A	2,412	0,772
Actin	46,696	32,174
Ratio	0,051653	0,0239945
Normalized ratio	1	0,5

Figure A2 (e)

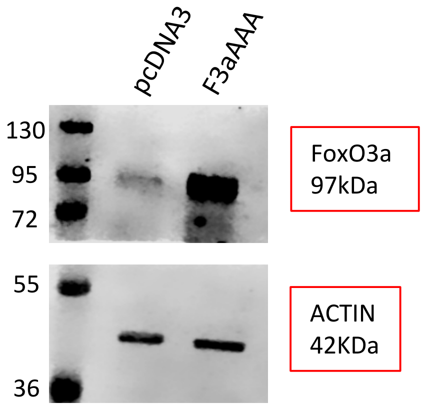

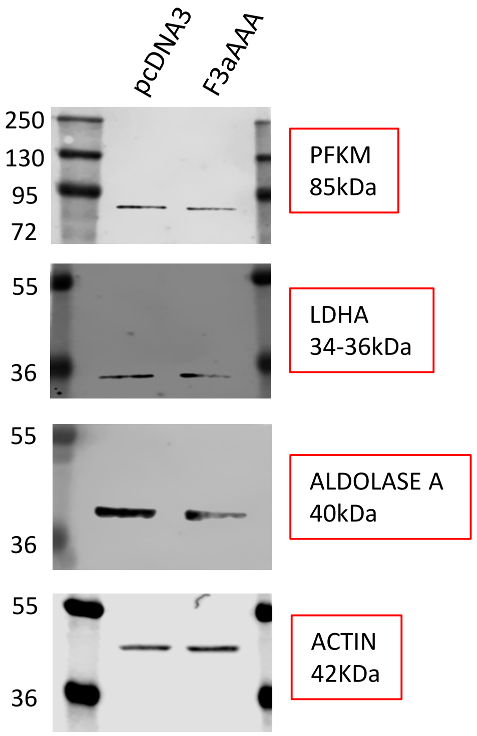

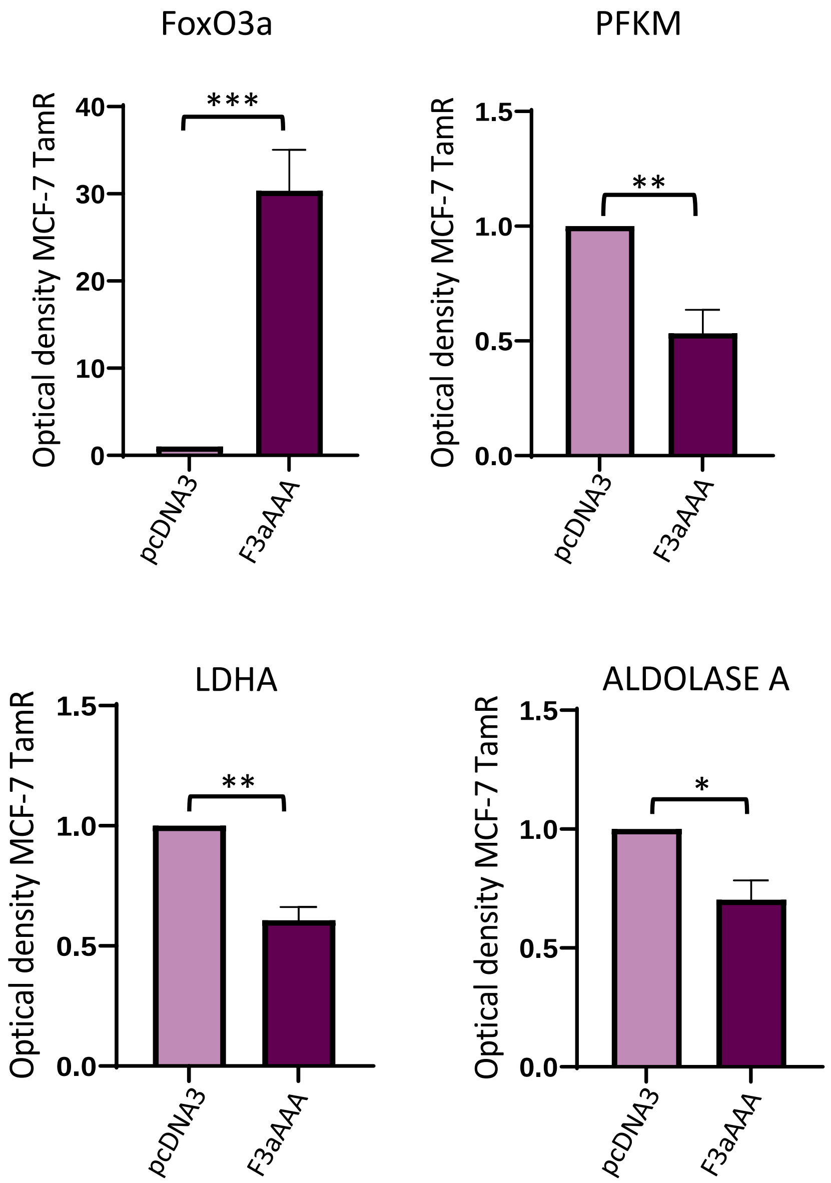

Lane	1	2
FoxO3a	0,0121	0,325
Actin	23,963	22,141
Ratio	0,0005	0,0146786
Normalized ratio	1	30

Lane	1	2
LDHA	0,763	0,684
Actin	3,985	6,365
Ratio	0,191468	0,1074626
Normalized ratio	1	0,6

Lane	1	2
PFKM	0,314	0,196
Actin	4,741	6,345
Ratio	0,066230	0,0308904
Normalized ratio	1	0,5

Lane	1	2
ALDOLASE A	3,712	3,105
Actin	4,745	6,053
Ratio	0,782297	0,5129687
Normalized ratio	1	0,7

Figure A2 (j)

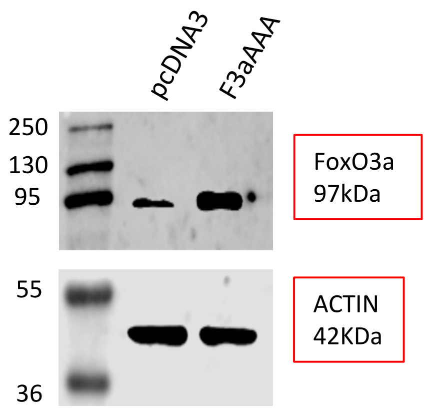

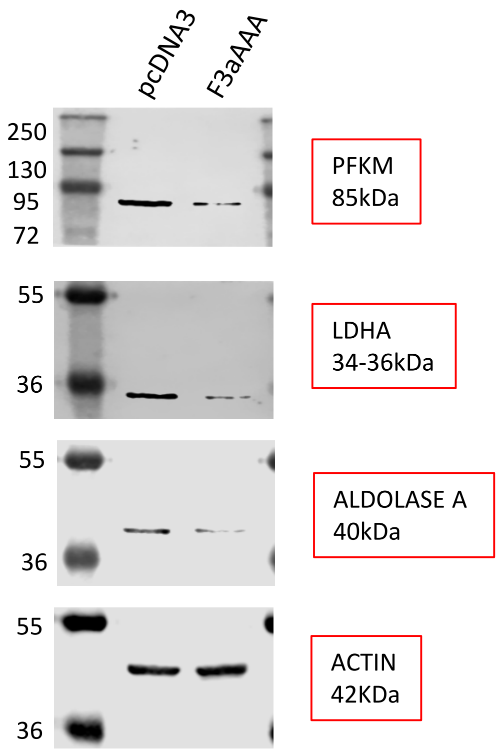

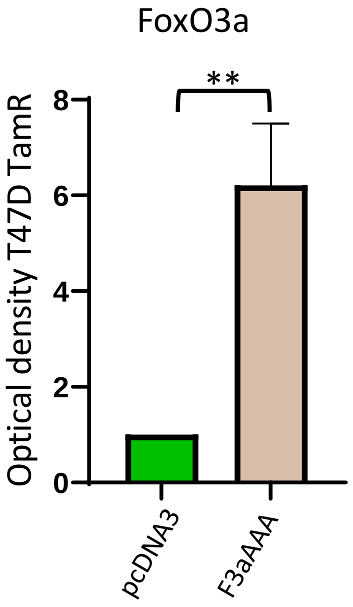

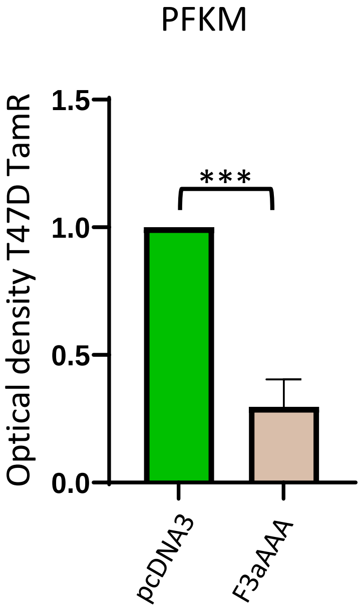

Lane	1	2
FoxO3a	0,385	3,169
Actin	4,126	5,456
Ratio	0,093310	0,5808284
Normalized ratio	1	6,2

Lane	1	2
LDHA	3,652	1,474
Actin	25,896	25,984
Ratio	0,141025	0,0567272
Normalized ratio	1	0,4

Lane	1	2
PFKM	1,632	0,448
Actin	25,985	26,126
Ratio	0,062805	0,0171476
Normalized ratio	1	0,3

Lane	1	2
ALDOLASE A	1,445	0,869
Actin	26,056	25,741
Ratio	0,055457	0,0337593
Normalized ratio	1	0,6

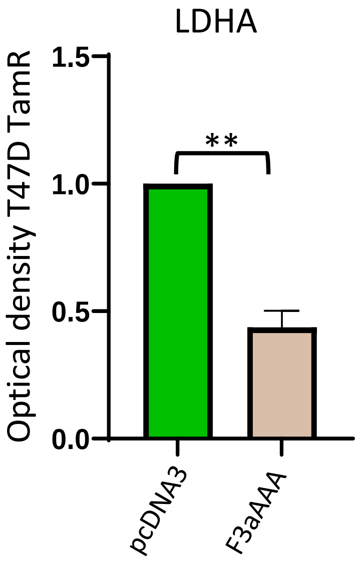

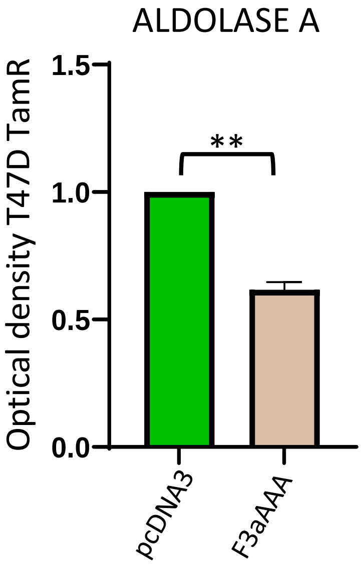

Figure A2 (o)

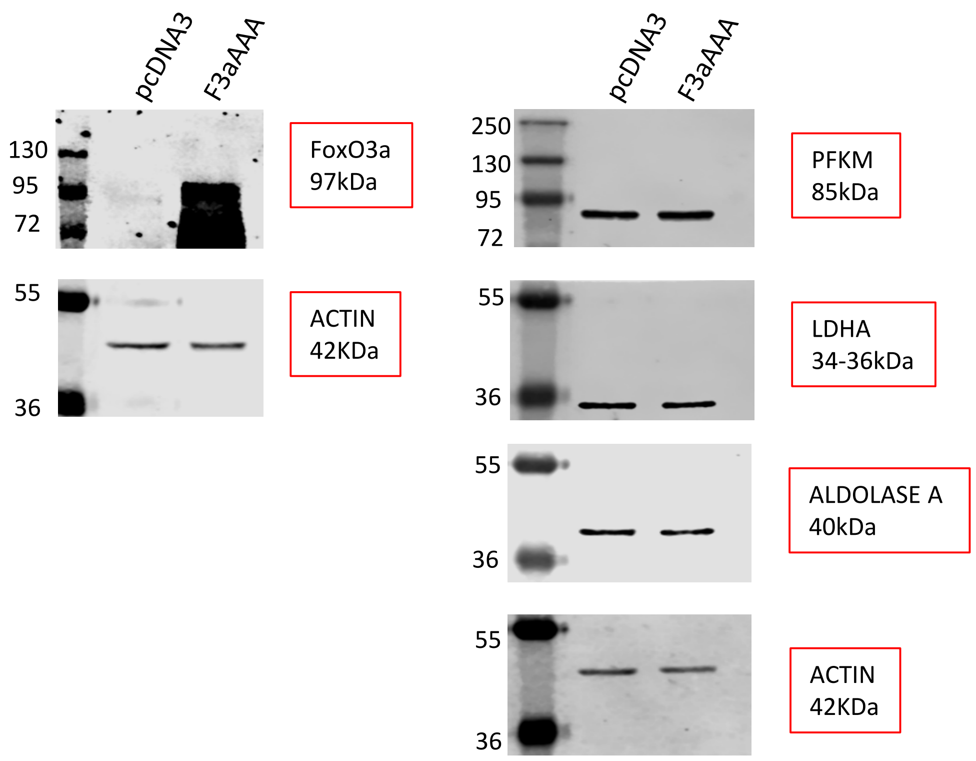

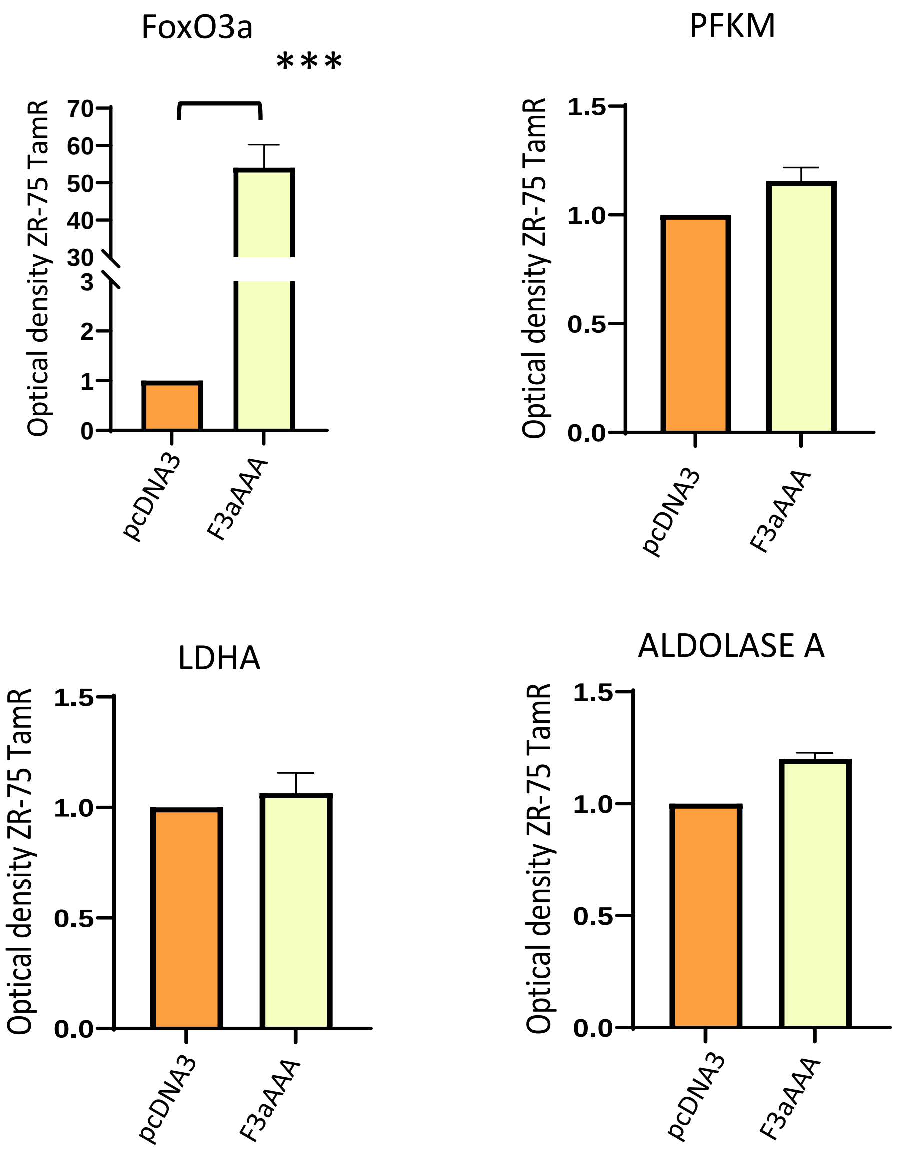

Lane	1	2
FoxO3a	0,0018	0,133
Actin	1,541	2,123
Ratio	0,001168	0,0626471
Normalized ratio	1	54

Lane	1	2
LDHA	1,052	1,101
Actin	2,125	1,974
Ratio	0,495058	0,5577507
Normalized ratio	1	1,1

Lane	1	2
PFKM	1,114	1,004
Actin	2,236	1,845
Ratio	0,498211	0,5441734
Normalized ratio	1	1,1

Lane	1	2
ALDOLASE A	4,256	4,496
Actin	2,744	2,434
Ratio	1,551020	1,8471651
Normalized ratio	1	1,2
